# Optimistic bias in updating beliefs about climate change longitudinally predicts low pro‐environmental behaviour

**DOI:** 10.1111/bjso.12905

**Published:** 2025-05-21

**Authors:** Tobias Kube, Jasmin Huhn, Claudia Menzel

**Affiliations:** ^1^ Department of Psychology University of Kaiserslautern‐Landau Kaiserslautern Germany

**Keywords:** belief updating, climate change, optimistic bias, pro‐environmental behaviour, risk perception

## Abstract

We investigated the preregistered hypothesis that an optimistic bias in updating beliefs about climate change (i.e., integrating good news more than bad news) cross‐sectionally (*N*_Study 1_ = 109) and longitudinally (*N*_Study 2_ = 407) predicts self‐reported pro‐environmental behaviour (PEB). To test this, we employed an experimental task in which participants were presented with multiple climate change scenarios and asked to update their beliefs after receiving scientific evidence. Additionally, we investigated whether biased belief updating and PEB could be altered by brief experimental interventions providing information on different aspects of climate change. Results show that optimistically biased belief updating did not predict PEB cross‐sectionally, but did predict PEB 4 weeks later, while controlling for baseline levels of PEB. The experimental interventions did not significantly alter belief updating or increase PEB, although there were significant gender differences. The results suggest that an optimistic bias in belief updating longitudinally predicts low engagement in PEB, possibly because selectively integrating good news over bad news reduces the perceived urgency to take action. Yet the effect may be small and detectable only in sufficiently large samples. The results also indicate that it is challenging to modify this bias. Implications for research on attitude change, social cognition and PEB are discussed.

## INTRODUCTION

When confronted with potential risks for their personal future, people usually prefer to integrate desirable information over undesirable information into their beliefs (Kube & Rozenkrantz, 2021; Sharot & Garrett, 2016). This valence‐dependent bias in information processing has been referred to as the ‘optimistic update bias’ (Garrett & Sharot, 2017; Moutsiana et al., 2015; Sharot et al., 2011). This means that people update their beliefs about their future more in response to information that is better than expected (i.e., ‘good news’) than in response to worse‐than‐expected information (i.e., ‘bad news’). Since climate change poses a broad range of risks to future prospects (Pörtner et al., 2022), research has begun to investigate whether the optimistic update bias also applies to the context of climate change.

A recent study presented participants in multiple trials with several risks related to climate change and examined how they adjusted their risk estimates in response to current scientific prognoses (Kube et al., 2024). The authors found that in the context of climate change, there was no significant optimistic update bias. That is, unlike the update of beliefs about their personal future prospects (Sharot et al., 2011), the majority of people did not integrate good climate news over bad climate news. The absence of the optimistic update bias in the context of climate change, as found by Kube et al. (2024), is also in line with other research showing that while people are optimistic about their personal future, they are less optimistic about global issues (Globig et al., 2022). However, an alternative interpretation of the results of Kube et al. (2024) may be that the optimistic update bias in the context of climate change is just smaller than in other contexts and can therefore only be found in sufficiently large samples. In addition, even if the effect was small, it may not be meaningless because Kube et al. also showed that those 35%–45% of participants who did integrate good news over bad news were less likely to endorse pro‐environmental attitudes and intentions to change their personal behaviour to mitigate climate change.

Therefore, one goal of the present research was to examine valence‐dependent belief updating in the context of climate change in a larger sample. In addition, since Kube et al. (2024) only focused on pro‐environmental intentions but not actual behaviour, it is unclear whether the propensity to integrate good news about climate change over bad news may also be associated with pro‐environmental behaviour (PEB). This distinction is important, as previous research has demonstrated a substantial gap between pro‐environmental intentions and actual behaviour, referred to as the intention‐behaviour gap (Bamberg & Möser, 2007; Gifford, 2011; Klöckner, 2013). Therefore, the present research aimed to examine whether optimistically biased belief updating in the context of climate change is associated with – and predictive of – PEB. Our hypothesis was that people who exhibit an optimistic bias in belief updating are less likely to engage in PEB. The underlying rationale was that a selective integration of positive climate‐related information at the expense of negative information may signal a cognitive avoidance of threatening environmental realities. By not sufficiently considering bad news about climate change, these individuals may experience lower perceived urgency or responsibility, ultimately reducing their motivation to adopt environmentally protective behaviours. In other words, when people systematically downplay climate risks, they may be less inclined to take action to mitigate them. To test this hypothesis, we examined the cross‐sectional (Study 1) and longitudinal (Study 2) relationship between belief updating and PEB.

In Study 2, we additionally investigated whether PEB can be enhanced through targeted experimental interventions that provide participants with information on different aspects of climate change. We reasoned that these interventions would influence how individuals update their climate‐related beliefs, fostering a more balanced integration of both positive and negative information. Given the expected link between optimistically biased belief updating and lower PEB, we hypothesised that participants in the experimental conditions would exhibit greater improvements in PEB over time, compared to those in the control condition.

Furthermore, we predicted gender differences in people's responses to the experimental interventions. Specifically, we hypothesised that women would increase their PEB more than men. This hypothesis draws from previous research showing that men are often more critical of climate change policies (Jylhä et al., 2016) and engage more in defensive self‐protective strategies than women (Wullenkord & Reese, 2021). Moreover, since women are often more responsive to threats (McClure et al., 2004; Ohrmann et al., 2010), we expected that the greater increase in PEB in women, relative to men, should be particularly pronounced if the intervention emphasizes the threats of climate change. This hypothesis is also supported by a previous study, which found gender differences in people's responses to the same experimental interventions as used in the current research, with men showing less increase in pro‐environmental intentions than women, particularly if the intervention emphasized threats (Kube et al., 2024). In sum, while the overarching goal was to examine the association between optimistically biased belief updating and PEB, the current research addressed the following four specific aims: to test whether

1. there is an optimistic update bias in the context of climate change,
2. optimistically biased belief updating longitudinally predicts low PEB,
3. optimistically biased belief updating and PEB can be altered through short experimental interventions,
4. there are gender differences in the effects of such interventions.

## STUDY 1

### Methods

#### Open Science statement

The main research question, hypotheses, study design, required sample size and analysis plan were prospectively preregistered: https://aspredicted.org/QLR_N4Z. The anonymised data set as well as the analysis code are publicly available: https://osf.io/vd4s8/?view_only=fd5e933e7526479cab8e96cd930fb04a. In this available data set, all sociodemographic variables were removed except for gender to ensure the anonymity of the participants. All studies, measures, manipulations and data/participant exclusions are reported in the manuscript or its Appendix S1.

#### Ethics statement

The study was approved by the local ethics committee of the Psychology Department of the University of Kaiserslautern‐Landau (reference number LEK_2020_276) and was conducted in accordance with the ethical standards as laid down in the 1964 Declaration of Helsinki and its later amendments.

#### Participants

The sample size was determined based on an a priori power analysis using G*Power 3.1. We based this power analysis on the linear regression analysis to determine the incremental effect of valence‐dependent belief updating in predicting pro‐environmental behaviour. Assuming a small to medium effect (*f* ^2^ = 0.08, *α* = .05, 1−*β* = .80, total number of predictors: 4), the a priori power analysis indicated a minimum sample size of 101 people.

A German convenience sample was recruited via email lists and flyers. A total of 186 people started working on the online study and 110 completed it. Of these, one person had to be excluded as a statistical outlier (>3 SD above the mean on the dependent variable), resulting in a final sample of 109 participants. The mean age of the sample was 33.4 years (SD = 15.3), 65.1% identified as females, 33.9% as males and 0.9% as diverse. The majority (55.0%) were university students. Data were collected online between August and October 2022.

#### Belief update task

The belief update task was originally developed in the context of people's personal future prospects (Sharot et al., 2011) and was subsequently adapted to the context of climate change (Kube et al., 2024). In this task, participants were presented with 20 scenarios, covering different adverse consequences of climate change (see Table S1). In each scenario/trial, participants first indicated how likely they think the adverse consequences mentioned in the scenario will occur (0%–100%; first estimation). Next, they were presented with the respective prognosis according to current scientific projections. Finally, participants responded again to the scenario by indicating a potential update of their personal estimation (i.e., second estimation) in light of the scientific prognosis received.

**FIGURE 1:**
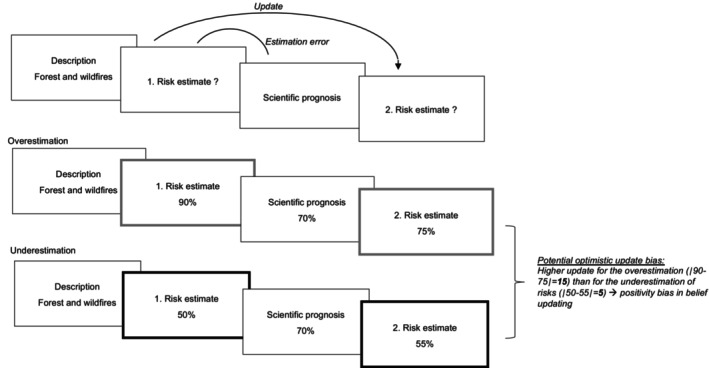
Illustration of the climate change‐related belief update task.

For each scenario, we calculated an estimation error (i.e., discrepancy between first estimation and prognosis). If the prognosis suggested a lower risk than participants initially expected, this information was considered ‘good news’, whereas a higher risk than expected was regarded as ‘bad news’ (see Figure 1). In each trial, we computed a belief update score, reflecting the absolute difference between participants' first and second estimation. In line with recent methodological recommendations (Sharot & Garrett, 2022), we divided each update score by the estimation error in the respective trial because greater discrepancies between participants' first estimations and actual prognoses will naturally result in greater belief updating. We then calculated the mean of these ‘normalised’ update scores separately for all trials in which a person received good news vs. bad news. The higher the update score for good news and bad news, respectively, the more people adjusted their initial risk estimation in response to the prognosis they were presented with. The difference between the update in response to good news and the update in response to bad news, which will be considered in many analyses presented below, is referred to as *asymmetry in belief updating*. In this respect, higher values reflect a greater update in response to good news than in response to bad news.

#### Pro‐environmental behaviour

Participants' pro‐environmental behaviour was assessed using the General Ecological Behaviour (GEB) scale (Kaiser et al., 2010, 2013; Kaiser & Wilson, 2004). The original GEB scale is composed of 50 statements on environment‐related behaviours from the factors (1) energy saving, (2) mobility, (3) waste avoidance, (4) consumption, (5) recycling and (6) social engagement. For the present study, the total number of items was reduced to seven items, which best reflect specific action plans to reduce the individual everyday impact on the environment in Germany (e.g., ‘In winter, I turn down my heating if I leave my home for more than 4 h; the list of all items can be found in the Appendix S1), according information from the company AT Kearney (Umweltbundesamt, 2021). The items were rated on a 5‐point scale (1 = *never*; 2 = *seldom*; 3 = *occasionally*; 4 = *often*; 5 = *very often*). When computing the sum score of the GEB‐7, we followed the recommendations of the scale developers to recode the frequency items into a dichotomous format by collapsing ‘never’, ‘seldom’ and ‘occasionally’ into ‘unreliable ecological engagement’, whereas ‘often’ and ‘very often’ were joined as ‘reliable ecological engagement’ (Kaiser et al., 2013; Kaiser & Wilson, 2004). Cronbach's alpha of the 7‐item GEB scale was .50.

#### Additional measures

Participants also completed measures of defensive self‐protective strategies, their perceived personal responsibility to mitigate climate change, their climate change‐related self‐efficacy and the perceived threat of climate change and sociodemographic basic variables, as detailed in the Appendix S1.

#### Statistical analyses

We conducted a paired *t*‐test to compare participants' belief update in response to good news vs. bad news. This analysis was not formally preregistered, but was necessary to examine whether there is an optimistic update bias in the climate task (Kube et al., 2024). Subsequently, we examined whether optimistically biased belief updating predicts PEB. To this end, we computed the variable *asymmetry in belief updating*, which reflects the difference between the update in response to good news vs. bad news (update_good news_ – update_bad news_), with values >0 reflecting a greater update in response to good news. To test whether this asymmetry in belief updating predicts PEB, we conducted a linear regression analysis with the sum score of the GEB scale as the dependent variable and asymmetry in belief updating as the predictor. Note that in the preregistration, we specified that we would also include self‐protection, responsibility and self‐efficacy as predictors, in addition to asymmetry in belief updating. This preregistered analysis is presented in the Appendix S1. Here, we decided to include only belief updating as a predictor to keep the focus of the overall paper clear. Type‐1 errors were set at 5%. All statistical analyses were conducted using IBM SPSS Statistics version 30.

### Results

#### Differences in belief updating towards good news vs. bad news

**FIGURE 2:**
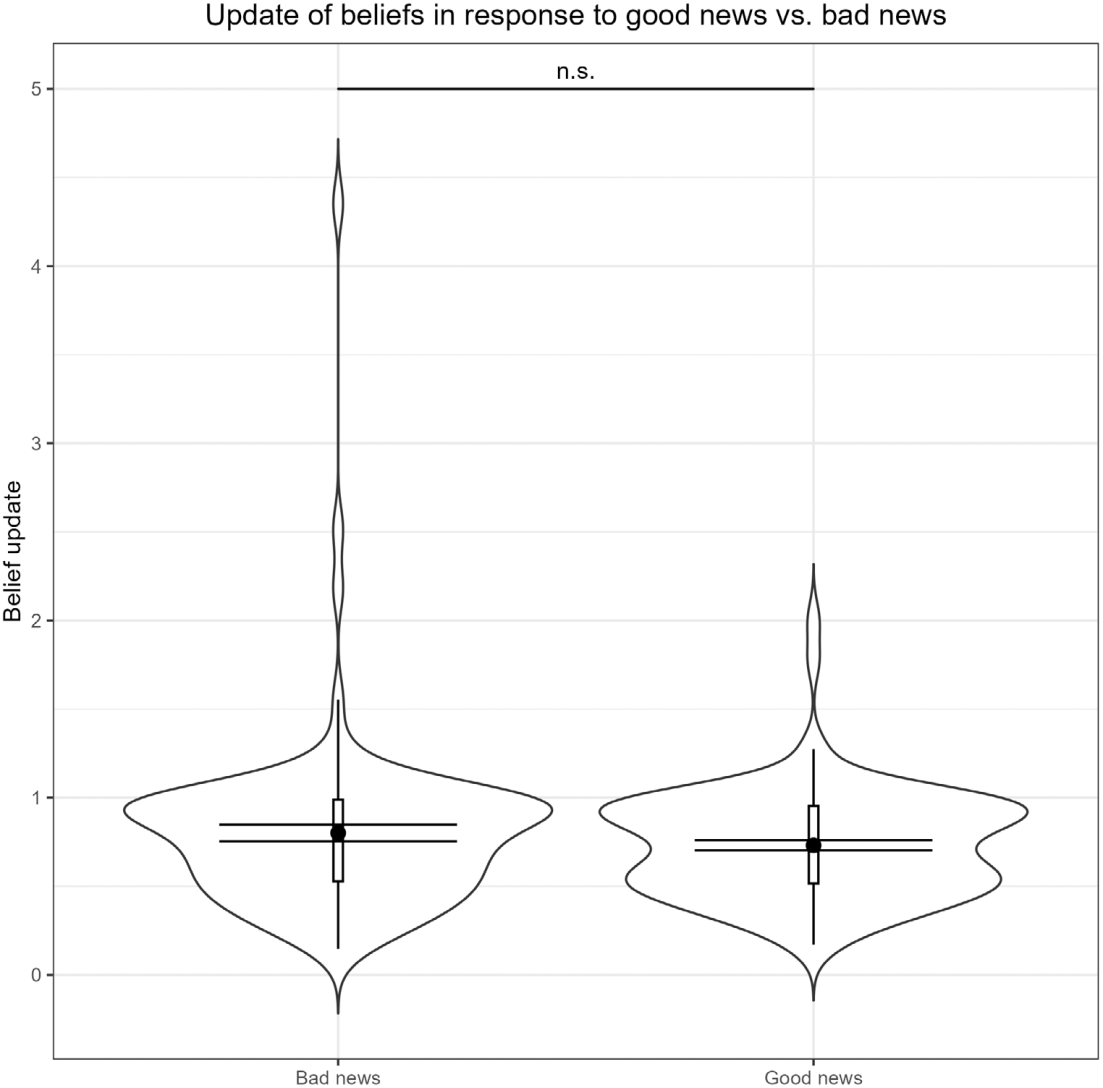
Violin plot to illustrate the results for belief updating in response to good news vs. bad news from Study 1. Error bars reflect 95% confidence interval.

The update of beliefs in response to good vs. bad news did not significantly differ, *t*(108) = −1.312, *p* = .192, *d* = −0.126, 95% CI [−0.314, 0.063], as illustrated in Figure 2. Without excluding the statistical outlier (see above), the difference was not significant either, *t*(109) = −1.069, *p* = .287, *d* = −0.102, 95% CI [−0.289, 0.086]. Putting it differently, 43.1% of the sample integrated good news over bad news, whereas 48.2% integrated bad news over good news (while the other 8.7% considered good news and bad news to the same extent).

#### Predicting pro‐environmental behaviour

Asymmetry in belief updating did not significantly predict PEB, *R* ^2^ = .002, *F*(1, 108) = 0.178, *p* = .674.

### Interim discussion

This study provided two main conclusions. First, the results show that the optimistic update bias (Sharot, 2011) was not found in the context of climate change, consistent with previous research (Kube et al., 2024). Second, the results of the regression analysis indicate that belief updating did not predict PEB cross‐sectionally.

## STUDY 2

The goal of this study was to extend the results from Study 1 by investigating how valence‐dependent belief updating is longitudinally associated with PEB, and to increase the robustness of the findings by examining a substantially larger sample. In addition, we aimed to investigate whether short experimental interventions, designed to inform people about different aspects of climate change, have the potential to modify valence‐dependent belief updating and, ultimately, lead to differences in PEB.

### Methods

#### Open Science statement

The research question, hypotheses, study design, required sample size and analysis plan of Study 2 were prospectively preregistered: https://aspredicted.org/MDS_ZX2. The anonymised data set, as well as the analysis code are publicly available: https://osf.io/vd4s8/?view_only=fd5e933e7526479cab8e96cd930fb04a. In the available data set, all sociodemographic variables were removed except for gender to ensure the anonymity of the participants. All studies, measures, manipulations and data/participant exclusions are reported in the manuscript or its Appendix S1.

#### Ethics statement

The study was approved by the local ethics committee of the university where the studies were performed (reference number LEK_2020_276) and was conducted in accordance with the ethical standards as laid down in the 1964 Declaration of Helsinki and its later amendments.

#### Participants

The required sample size was determined based on an a‐priori power analysis, which focused on the study's aim to test the effects of the three experimental interventions on PEB in comparison to a control video. We based our power analysis on this aim because we expected this effect to be the smallest among the above‐mentioned effects of interest, with reference to previous research using the same experimental interventions (Kube et al., 2024). Accordingly, the power analysis using G*Power for a repeated‐measures ANOVA (expected *f* = 0.10; *α* = .05; 1−*β* = .80) indicated a minimum sample size of 280 participants. Yet, we aimed to exceed this suggested minimum sample size to increase the degree of certainty of the results. Specifically, we aimed to have a final sample size of about 400 participants at follow‐up, thereby following recent recommendations to have at least 100 participants per experimental condition. Participants were recruited over 2 years in Germany between February 2022 and February 2024 via email lists, flyers and postings in social networks.

A total of 864 people began to work on the study at baseline, of whom 721 completed it. Of these, four people were younger than 18 and therefore excluded. Based on the preregistered criteria for excluding cases, one person had to be excluded as a statistical outlier (>3 SD above the mean in belief updating), and 112 participants were excluded because they did not endorse a variety of control items, which were designed to ensure the quality and trustworthiness of data collected online. Thus, 604 participants were used for the analysis sample at baseline, of whom 407 participants also completed the follow‐up assessment 4 weeks later. From the initial *N* = 604 sample, 74.8% identified themselves as females, 24.8% as males and 0.4% as diverse. The mean age in the present sample was M = 26.63 (range 18–84, SD = 9.60). Most people had school leaving examination (German ‘Abitur’ or ‘Fachabitur’, 57.9%) or a university degree (38.4%) as the highest educational degree, while 1.8% had primary education, 0.7% were still at school and 1.2% indicated having another (unspecified) educational degree.

#### Procedure

After providing informed consent, participants first reported on their current PEB before completing a questionnaire on their intentions to engage in more PEB. Subsequently, they indicated how threatening and anxiety‐provoking they perceived climate change overall and for them personally. Afterwards, participants completed the 20‐trial belief update task as described for Study 1 After the first 10 trials, participants were randomly assigned to one of the four experimental conditions, each presenting participants with a different video (see below). Next, they completed the second half of the belief update task and subsequent brief questionnaires. Finally, they were informed about receiving the link for the second part of the study 4 weeks later via email.

#### Experimental manipulations of pro‐environmental behaviour

For the three experimental interventions, we used the same video‐based approach as a previous study to ensure comparability (Kube et al., 2024). Thus, participants were presented with short video clips informing about different aspects of climate change. The videos of the three experimental conditions were produced by the German TV broadcast ‘Quarks & Co.’ to ensure that the videos were comparable in terms of style and quality. Participants who were allocated to the condition called *Threat* were shown a video, in which the threatening consequences of climate change are illustrated (from min 0:02 to 2:48). Participants from the *Threat + Options for Action* condition first watched the same video as the *Threat* condition; subsequently, they were shown another Quarks & Co. video emphasizing individual options to reduce greenhouse gas emissions (from min 0:00 to 3:09). Finally, participants from the *Psychoeducation* condition participants watched a Quarks & Co. video, which explained that most people are prone to certain biases in their personal risk perception, including the underestimation of risks when their consequences are expected to occur in the more distal future as opposed to the proximal future, as can be observed in how people deal with the risks of climate change (from min 20:20 to 24:33). In addition, participants watched a sequence of a Ted Talk by Tali Sharot, in which she spoke about the optimistic updated bias in particular (https://www.youtube.com/watch?v=B8rmi95pYL0&t=19s; from min 0:15 to 2:38). Participants from the control group watched a sequence from the film ‘Bend it like Beckham’, which is about two girls who dream of playing football (from 67:28 to 71:34 min). It was selected because the control video was to be independent of topics related to climate change.

The rationales for the three different intervention approaches were drawn from some specific lines of research. Specifically, with reference to research on ‘fear appeals’ (Ruiter et al., 2014; Witte & Allen, 2000), we tested the effects of an intervention that informed people about the threatening consequences of climate change (experimental condition referred to as ‘Threat’). The rationale was that emphasising the threatening consequences of climate change if necessary action is not taken would lead to greater integration of bad news, thereby increasing the likelihood that people would engage in PEB. In another experimental condition (referred to as ‘Threat + Options for Action’), we drew from more recent work to suggest that only informing people about potentially threatening consequences is not sufficient for behaviour change, but people also need specific alternative behavioural options (Bieniek‐Tobasco et al., 2020; Carlson et al., 2020; Nisa et al., 2019). In the third condition (referred to as ‘Psychoeducation’), we shared knowledge about typical biases in human risk perception with participants (including the optimistic update bias). Appealing to research from experimental psychopathology (Kube et al., 2019), we investigated whether such an educational approach would lead to a greater integration of bad news and, as a result, more engagement in PEB.

#### Measures

##### Pro‐environmental behaviour

Because of the poor psychometric properties of the short version of the GEB, as used in Study 1, we decided to use the full 50‐item General Ecological Behaviour (GEB) scale (Kaiser et al., 2010, 2013; Kaiser & Wilson, 2004) in Study 2. Of the total 50‐item scale, 32 items assess how frequently participants engage in various pro‐environmental actions, using a 5‐point scale (1 = *never*; 2 = *seldom*; 3 = *occasionally*; 4 = *often*; 5 = *very often*). Another 18 items assess dichotomously whether or not they engage in different pro‐environmental actions. Participants are instructed to refer to the last 4 weeks when indicating how frequently they engaged in the various pro‐environmental actions.

Nineteen items, which were negatively formulated, were reversely scored, such that for the resulting total score, which ranges between 1 and 2, higher values indicate more pro‐environmental behaviour. When computing the sum score of the GEB‐50, we followed the recommendations of the scale developers to recode the 32 items that assess the frequency of behaviours into a dichotomous format by collapsing ‘never,’ ‘seldom,’ and ‘occasionally’ into ‘unreliable ecological engagement’, whereas ‘often’ and ‘very often’ were joined as ‘reliable ecological engagement’ (Kaiser et al., 2013; Kaiser & Wilson, 2004). The GEB‐50 total score at baseline correlated significantly with the total score 4 weeks later, *r* = .604, *p* < .001. At baseline, Cronbach's *α* was .72 and at follow‐up it was .73.

##### Other measures

In addition, we assessed people's intentions for PEB and the presence of depressive symptoms among participants. These measures and the corresponding results are presented in the Appendix S1.

#### Statistical analyses

As in Study 1, we first conducted a paired *t*‐test to compare participants' belief update in response to good news vs. bad news. As preregistered, we used only the data from the first ten trials for this comparison, because trials 11–20 might have been affected by the experimental interventions. Second, we examined whether asymmetric belief updating (i.e., computing the difference score: update_good news_ – update_bad news_ as in Study 1) longitudinally predicts PEB in a non‐preregistered hierarchical linear regression analysis. In this regression analysis, PEB at baseline was entered as a predictor first, and the asymmetry in belief updating second. Third, to examine the effects of the experimental interventions, we first examined in a time (pre‐intervention vs. post‐intervention) by condition (*Threat* vs. *Threat + Options for Action* vs. *Psychoeducation* vs. control group) repeated‐measures ANOVA whether the four experimental conditions differ in changing valence‐dependent belief updating. Next, we performed the same 2 by 4 ANOVA, but this time with the GEB sum score as the dependent variable, as preregistered. With respect to the latter, we also examined the modulating effects of gender by subsequently adding gender (male vs. female) as an additional between‐subjects factor, as preregistered. We did not consider people who indicated a non‐binary gender in this analysis, because too few people indicated it to be meaningfully (and statistically robustly) examined. In the supplement, we also report results of additional effects of the experimental interventions on pro‐environmental intentions and the accuracy of risk perception. Type‐1 errors were set at 5%. All statistical analyses were conducted using IBM SPSS Statistics version 30.

### Results

#### Differences in belief updating towards good news vs. bad news

**FIGURE 3:**
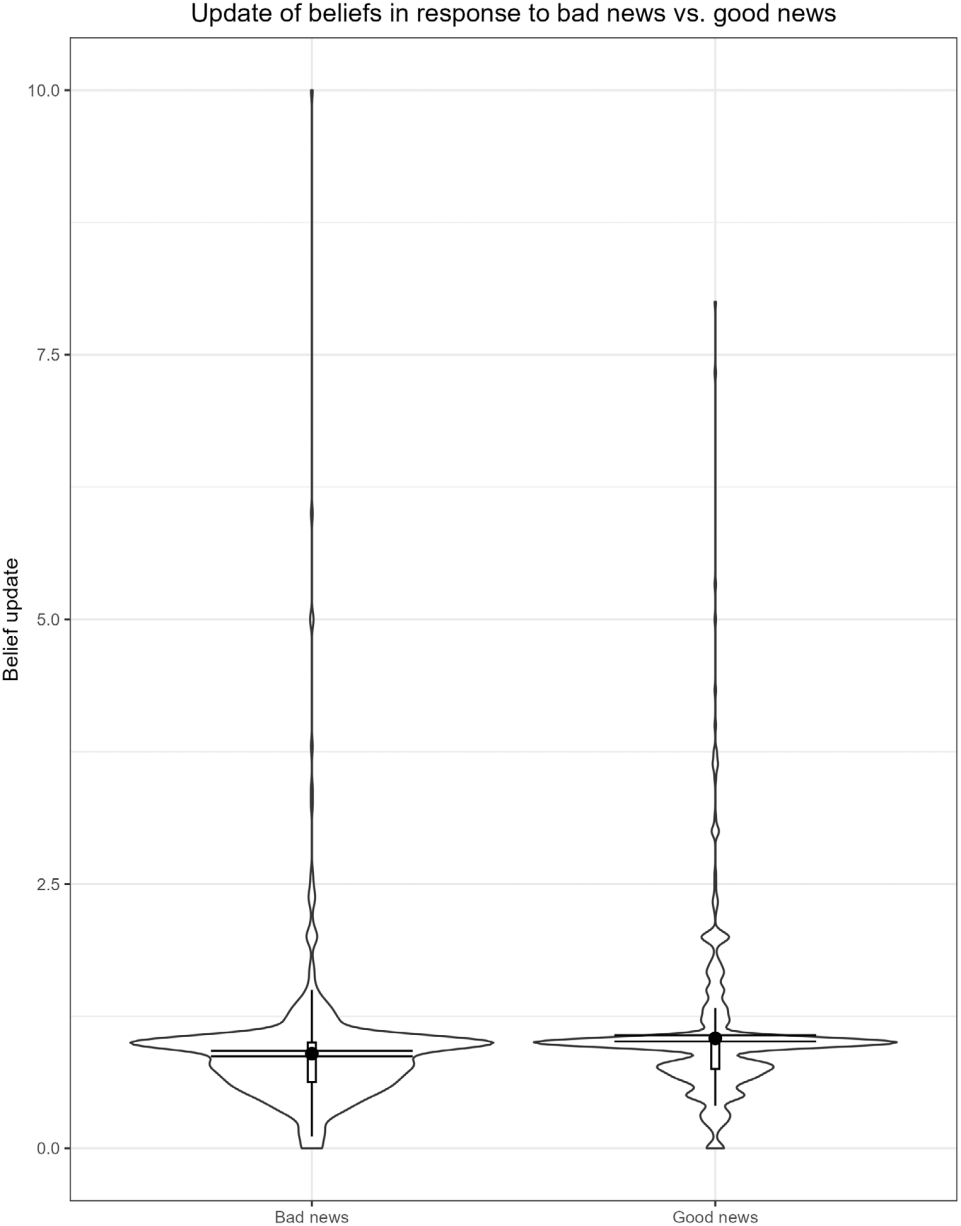
Violin plot to illustrate the results for belief updating. Error bars reflect 95% confidence interval. The update in response to good news was significantly greater than in response to bad news, but the effect size was very small *d* = 0.160, 95% CI [0.080, 0.241].

Participants updated their beliefs significantly more in response to good news than in response to bad news, *t*(603) = 3.941, *p* < .001, *d* = 0.160, 95% CI [0.080, 0.241], reflecting a small effect, as illustrated in Figure 3.^2^ Putting it differently, 47.2% integrated good news over bad news, whereas 32.7% integrated bad news over good news (while the other 20.1% integrated it equally).

#### Predicting environmental behaviour

The results show that PEB at baseline significantly predicted PEB 4 weeks later, *R* ^2^ = .407, *F*(1, 405) = 278.413, *p* < .001. Adding the asymmetry in belief updating (trials 1–10) as a predictor in the second step added another 1% of explained variance, which was a significant increase, *R* ^2^ = .415, *F*(1, 404) = 7.175, *p* = .008. Integrating good news over bad news was associated with less PEB 4 weeks later (*β* = −.102, *p* = .008).

#### Effects of the experimental interventions on belief updating

A repeated‐measures ANOVA indicated a significant main effect of time, *F*(1, 592) = 11.799, *p* < .001, *η*_p_^2^ = 0.020, 95% CI [0.004, 0.047], showing that across conditions, participants' tendency to integrate good news over bad news significantly declined from the pre‐intervention trials (1–10) to the post‐intervention trials (11–20), resulting in a more balanced integration of good news and bad news. The time by condition interaction was not significant, *F*(3, 592) = 1.857, *p* = .136, *η*_p_^2^ = 0.009, 95% CI [0, 0.026], although descriptively the decline was slightly higher in the *Threat* condition and the *Threat + Options for Action* condition than in the control condition and the *Psychoeducation* condition (see Figure S1).

#### Effects of the experimental interventions on pro‐environmental behaviour

The repeated‐measures ANOVA indicated that neither the main effect of time, *F*(1, 403) = 2.782, *p* = .096, *η*_p_^2^ = 0.007, 95% CI [0, 0.032], nor the main effect of condition, *F*(3, 403) = 0.572, *p* = .634, *η*_p_^2^ = 0.004, 95% CI [0, 0.018], nor the time by condition interaction was significant, *F*(3, 403) = 0.362, *p* = .780, *η*_p_^2^ = 0.003, 95% CI [0, 0.013].

**FIGURE 4:**
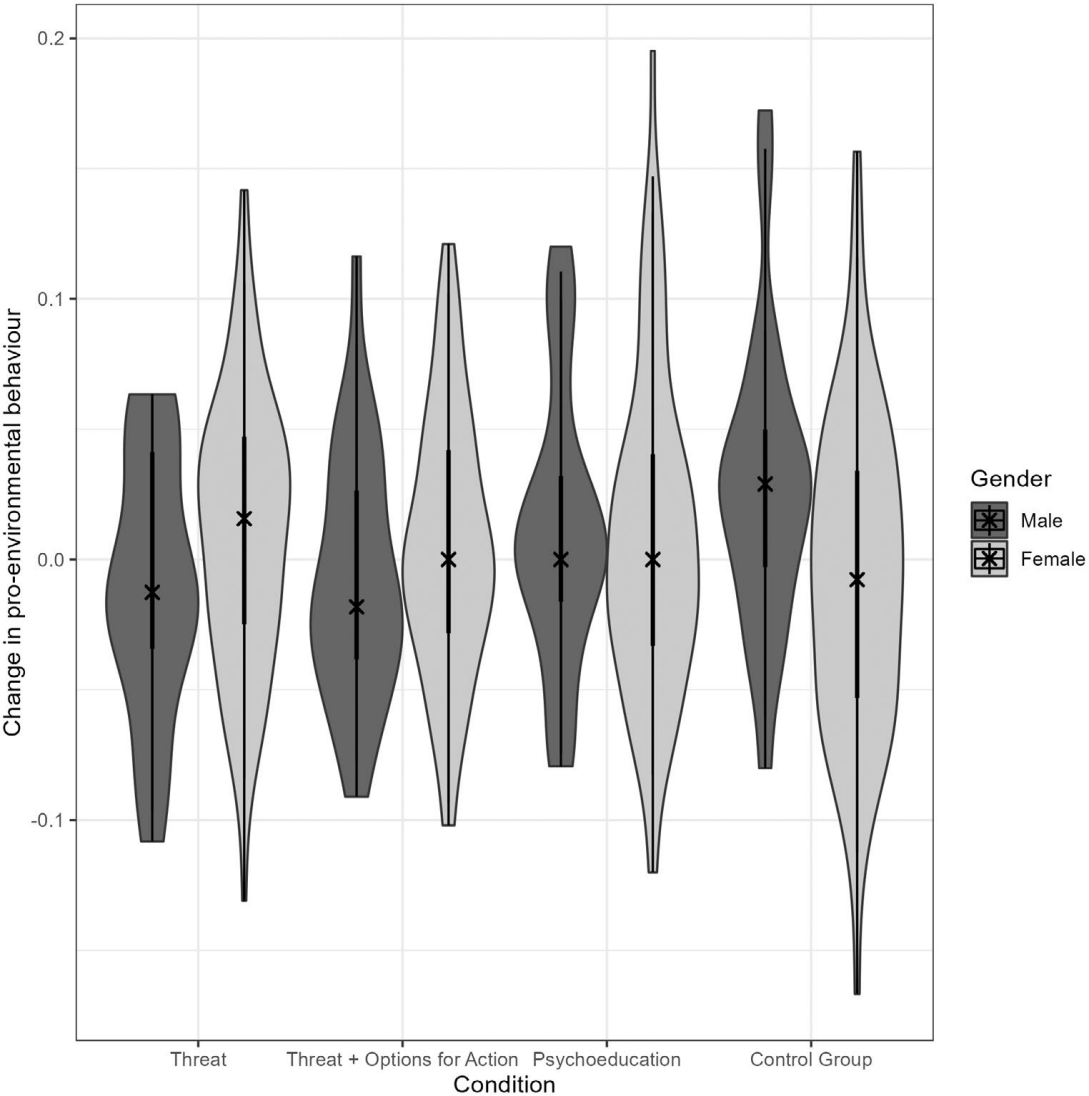
Violin plot for gender differences in changes in pro‐environmental behaviour. Higher values reflect a greater increase in pro‐environmental actions at the 4‐week follow‐up as compared to baseline. The × reflects the median. Results show that in conditions in which a video about the threats of climate change was shown, men, unlike women, even somewhat lowered their pro‐environmental actions. Surprisingly, men showed the greatest increase in pro‐environmental actions in the control group, which did not receive any specific information on climate change.

However, when adding gender as another between‐subjects factor, a significant time by condition by gender interaction emerged, *F*(3, 399) = 3.503, *p* = .016, *η*_p_^2^ = 0.026, 95% CI [0.001, 0.058]. As can be seen in Figure 4, this three‐way interaction reflects that in the two conditions where participants were informed about the threatening consequences of climate change (i.e., *Threat* and *Threat + Options for Action*), women increased their PEB from baseline to 4 weeks later, whereas men in this condition *lowered* their PEB in that period, *t*(220) = −1.992, *p* = .048, *d* = −0.325, 95% CI [−0.645, −0.003]. By contrast, in the control condition, men, unlike women, significantly increased their PEB, *t*(87) = 2.446, *p* = .016, *d* = 0.584, 95% CI [0.107, 1.059]. A significant main effect of gender indicated that women showed overall more PEB than men, *F*(1, 399) = 7.946, *p* = .005, *η*_p_^2^ = 0.020, 95% CI [0.002, 0.054].

### Interim discussion

Unlike Study 1 and previous research (Kube et al., 2024), participants from Study 2 updated their beliefs about climate change more towards good news than towards bad news, thus speaking to the original optimistic update bias (Sharot et al., 2011). This significant effect was most likely revealed through the large sample size and thus more statistical power. In Study 2, we also found that an optimistic update bias predicted low PEB 4 weeks later, while controlling for baseline levels of PEB. Regarding the effects of the experimental interventions, the results show that they could not decrease optimistically biased belief updating and increase PEB significantly more than the control condition. However, the preregistered analyses regarding gender confirmed that the effects of the experimental interventions on PEB differed between women and men, while women showed overall more engagement in PEB than men.

## GENERAL DISCUSSION

The primary goal of the present research was to investigate whether an optimistic bias in updating beliefs about climate change predicts PEB. To pursue this goal, we first examined whether there is an optimistic bias in belief updating. The results of Study 1 indicated none, whereas Study 2, using a substantially larger sample, did find an optimistic update bias. Second, we investigated whether people who show the optimistic update bias are less likely to engage in PEB. In the cross‐sectional analysis of Study 1, we did not find this association, but in the longitudinal analysis of Study 2, we did find that the preference to integrate good news over bad news predicted lower PEB, above and beyond baseline levels of PEB.

The present findings extend our knowledge about valence‐dependent information processing as they show that the well‐known optimistic bias in belief updating (Sharot et al., 2011) may also apply to the context of climate change, according to the results from Study 2. However, since the effect size was small, the results suggest that this effect can only be found in a sufficiently large sample. This suggestion is consistent with the fact that both Study 1 and previous research, both of which examined smaller samples (Kube et al., 2024), did not find this effect. Our corresponding correlational analyses, reported in the supplement, speak to the interpretation that the propensity to integrate good news over bad news in the context of climate change may be smaller compared to other contexts because climate change is related to a sense of threat, as also demonstrated by previous research which manipulated the sense of threat experimentally (Garrett et al., 2018).

Although the optimistic update bias in the context of climate change is small, it may not be meaningless, as the results of the longitudinal analyses point to its predictive value for PEB. Specifically, we found that optimistically biased belief updating predicted less PEB 4 weeks later. As a potential mechanism of this association, we suggested that the integration of good news over bad news may reflect the cognitive avoidance of negative information and thus reduce the perceived urgency to take personal action to mitigate climate change. Yet, this proposed underlying mechanism cannot be further elucidated with the current data and must therefore be investigated further in future research. In addition, it must be noted that the association of belief updating and PEB was rather small and it is unclear how much belief updating would add if other psychological predictors were included.

An additional goal of the present research was to examine whether short video‐based interventions can foster a more balanced integration of both good news and bad news and increase people's engagement in PEB. In sum, similar to previous research (Kube et al., 2024), we found the effects of the currently investigated interventions to be small and non‐significant. Future research may therefore need to use other contents for interventions designed to enhance PEB. To this end, future research may consider the findings from a large multi‐national investigation in which several psychological climate interventions were examined (Berkebile‐Weinberg et al., 2024; Vlasceanu et al., 2024).

When testing the effects of the current interventions – focusing on the threatening consequences of climate change, individual options for action and human biases in risk perception – we found gender differences in people's responses to them, similar to previous research (Kube et al., 2024). Specifically, the results show that in response to the conditions in which the threatening consequences of climate change were emphasized, women increased their PEB, whereas men decreased it. By contrast, women showed no increase in PEB in the control condition, which watched a video unrelated to climate change, whereas men surprisingly increased their PEB in that condition. We believe that these findings are interesting and relevant to climate change communication and therefore we elaborate a bit more on them.

One possible interpretation of the gender differences in response to the intervention videos is that men, more than women, show psychological reactance (Brehm & Brehm, 2013) when being informed explicitly about the threatening consequences of climate change and the necessity to change the way they live. Thus, if men feel pushed into certain actions, they might react defiantly – possibly due to a perceived threat of losing lifestyle aspects socially associated with masculinity (Swim et al., 2020). A possible interpretation of the increase in PEB in men in the control condition may be that the lack of a specific instruction made it easier for men to critically reflect on their own role and thus come to the conclusion by themselves that more needs to be done to mitigate climate change. However, the current data does not allow testing specific interpretations of this effect. Therefore, we encourage future research to look into these gender differences in greater depth. This could be valuable not only because we also found gender differences in other variables reported in the Appendix S1, but also because previous research consistently pointed to gender differences in people's perception of – and response to – climate change (Bloodhart & Swim, 2020; Kube et al., 2024; McCright & Dunlap, 2011).

### Implications

The current findings have some implications for research on important areas of research in social psychology. First, the finding that good news about climate change is integrated more than bad news (provided that a sufficiently large sample is examined, as in Study 2) is in line with research on attitude change and motivated cognition (Ditto & Lopez, 1992; Kunda, 1990; Taber & Lodge, 2006), as it suggests that information processing is not neutral but guided by factors such as desirability. Relatedly, the current results can be linked to research on solution aversion, which proposes that people reject evidence that implies solutions they find undesirable—e.g., if accepting climate change requires them to support policies they oppose, they may downplay the severity of climate change (Campbell & Kay, 2014). Additionally, the results can be related to pro‐social behaviour research, as the finding that an optimistic update bias longitudinally predicted lower PEB suggests that insufficient consideration of undesirable information may diminish people's sense of urgency or personal responsibility. This aligns with research on diffusion of responsibility, where people are less likely to act when they believe the situation is under control or being managed by others (Darley & Latané, 1968; Garcia et al., 2002; Gifford, 2011). Furthermore, the results regarding the experimental interventions, showing gender differences in the responses to threat‐based information, fit with broader findings in social cognition indicating that men and women may interpret and react to social and environmental threats differently (Brough et al., 2016; Kahan et al., 2017; Lerner et al., 2003). These findings may also offer potential for further research into whether climate change communication can be improved by tailoring it to gender‐specific messages.

### Limitations

In our view, our work provides valuable insights into how people process climate change forecasts and how valence biases in this respect predict the engagement in PEB. Further strengths of the present research can be seen in the use of a belief update task, which was adapted to the context of climate change previously (Kube et al., 2024), the assessment of PEB beyond pro‐environmental intentions, the use of both cross‐sectional and longitudinal data, preregistrations and the transparency regarding data and materials.

However, an important limitation is that our samples were relatively young, largely female, little diverse and highly educated. Related to the samples, we note that the gender differences must be interpreted cautiously, given the unequal distribution of female and male participants. Accordingly, future research that seeks to systematically follow up on the gender differences from the present work may wish to aim for an equal distribution of male and female participants. Regarding the assessment of PEB, we note that although superior to only asking about intentions for PEB, the GEB‐50 is still a self‐report questionnaire and as such susceptible to biases such as social desirability. Therefore, future research may consider the use of behavioural tasks to assess PEB beyond self‐report (Berger & Wyss, 2021; Lange et al., 2018; Lange & Dewitte, 2021).

## CONCLUSIONS

The present research investigated how people update their beliefs about climate change in response to climate change forecasts. We found a significantly greater update in response to good news, yet with a small effect size, and this effect could not be found in another independent study using a smaller sample. Optimistically biased belief updating predicted low PEB 4 weeks later, but again this effect could not be found in cross‐sectional analysis using a smaller sample. Together, these findings suggest that optimistically biased belief updating may be an important predictor of PEB, but this effect may be small and detectable only in sufficiently large samples. Future research may aim to investigate the underlying mechanisms of the longitudinal association between optimistically biased belief updating and PEB, potentially focusing on the role of perceived urgency.

## AUTHOR CONTRIBUTIONS

**Tobias Kube:** Conceptualization; investigation; writing – original draft; methodology; validation; visualization; writing – review and editing; software; formal analysis; project administration; resources; supervision; data curation. **Jasmin Huhn:** Conceptualization; investigation; methodology; writing – review and editing. **Claudia Menzel:** Conceptualization; methodology; writing – review and editing; project administration; supervision.

## FUNDING INFORMATION

The present research received no funding.

## CONFLICT OF INTEREST STATEMENT

The authors have no conflict of interest to declare.

## ETHICS STATEMENT

The study was approved by the local ethics committee of the university where the studies were performed (reference number LEK_2020_276) and were conducted in accordance with the ethical standards as laid down in the 1964 Declaration of Helsinki and its later amendments.

## Supporting information


Appendix S1

## Data Availability

The anonymised data seta as well as the analysis codes are publicly available: https://osf.io/vd4s8/?view_only=fd5e933e7526479cab8e96cd930fb04a.

## Footnotes

1 A novel feature of the update task used in this study is that participants indicated for each of the 20 climate change events also as how threatening and anxiety‐provoking they perceive it. The preregistered analyses regarding the association of this threat perception and anxiety provocation with belief updating are reported in the supplement.

^2^
Without excluding cases (as described above), there was also significantly greater belief update in response to good news than in response to bad news, *t*(790) = 4.065, *p* < .001, *d* = 0.145, 95% CI [0.074, 0.215].
