# Supplementary material for: Optimistic bias in updating beliefs about climate change longitudinally predicts low pro‐environmental behaviour
Source: Br J Soc Psychol. 2025 May 21;64(3):e12905. doi: 10.1111/bjso.12905 (PMC12095902; doi:10.1111/bjso.12905)
Supplement: Supplementary file 1 — Appendix S1 [file BJSO-64-0-s001.docx]

**Supplementary Materials**

**General Material**

Table S1

*List of climate scenarios*

| **Scenario title** | **Introduction** | **Prognosis** |
| --- | --- | --- |
| Thawing permafrost soils | A quarter of the Earth's land surface consists of permafrost soils. They store large quantities of carbon dioxide (CO2) and methane are stored in them. Due to global warming these areas are melting and the climate-damaging greenhouse  greenhouse gases are released into the atmosphere, accelerating the rate of global warming. | According to an estimate by the Intergovernmental Panel on Climate Change for a pessimistic climate model, **81%** of the surfaces of the permafrost could be thawed by 2100. The release of the greenhouse gases stored in it would lead to an acceleration of global warming, which would also trigger droughts, forest fires, heavy precipitation events, floods and other extreme weather events and natural disasters in Germany (Brasseur et al., 2017; Knauer, 2019; Stocker, 2014). |
| Economic downfall due to climate-related catastrophes | Climate change is a global phenomenon that will affect the entire world economy. Even in an optimistic scenario, scientists expect that per capita economic output will be significantly reduced by the end of the century. | Due to its high share of exports to all countries of the world, Germany is highly dependent on the global economy. Compared to a development without climate change, global per capita income would drop by **23%** by 2100. In Germany, the economic consequences would arise, among other things, through international feedback processes. For example, water scarcity in South Korea can affect the prices of agricultural products in Germany (Brasseur et al., 2017; Burke et al. 2015). |
| Droughts in Germany | Droughts are characterised by long-lasting high temperatures and relatively little precipitation. This results in an extraordinary water deficit in the soil over a large area, so that an adequate water supply for the plants is not guaranteed. | The average drought duration in Germany will increase by **50%** with a global warming of 3°C compared to the period 1971-2000 (Thober et al., 2018). The German Meteorological Service warns of the consequences of droughts for agriculture, energy supply and inland navigation as well as of an increased risk of forest fires. |
| Forest dieback in Germany | A large part of the land mass is covered by forests. They extract carbon dioxide (CO2) from the atmosphere, e.g., through photosynthesis. Carbon dioxide is a greenhouse gas that contributes to the warming of the earth. Due to the massive disruption of forest systems caused by climate change, humanity is losing important resources in the reduction of carbon dioxide (CO2). For example, spruce stands, which make up a large part of Germany's forests, will decline sharply. Forest dieback will also bring further disadvantages for individuals in Germany. | Almost one third of Germany is covered with forest. Recent years have made it clear that Germany's forests are poorly able to adapt to changing climatic conditions. According to calculations, in a pessimistic climate model the suitable area for spruce cultivation could decrease by **68%** in the federal state of Baden-Württemberg by 2065. Spruce alone accounts for more than a quarter of the German forest stand. Forests have been shown to reduce stress and have a positive effect on health. These and other benefits would be lost if forest dieback in Germany is not curbed (Hanewinkel et al., 2010; James et al., 2015; Ulrich et al., 1991). |
| Global warming and violence | Global warming will lead to resource scarcity and forced migration of people living in regions affected by natural disasters, for example. For this reason, among others, a connection between global warming and the number of armed conflicts can already be established today. Forecasts assume that the number of wars will continue to rise drastically in the future, depending on global warming. | With a temperature rise of 4°C, the number of armed conflicts could increase by about **50%.** In addition to wars, violence among people would also very likely increase as a function of global warming. In the USA alone, for every 1°C rise in temperature, 24,000 more violent attacks are expected each year ((Doherty & Clayton, 2011; Hsiang et al., 2013). |
| Climate refugees | A continuing rise in temperature and increasing extreme weather conditions and natural disasters in large parts of the world mean that many people have to leave their homes in order to save their livelihoods. The number of so-called "climate refugees" in Europe and Germany will continue to increase in the future. | With a temperature increase of 4°C, scientists predict a risk of about **70%** for displacement due to extreme weather events between 2080 and 2100. The World Bank expects about 140 million "climate refugees" worldwide by 2050. Already in 2019, almost half of Germans thought that Germany had overstretched itself in taking in refugees and should not take in any more refugees for the time being. The proportion of refugees due to the consequences of climate change would be many times higher than in the situation at that time (Ahsan et al., 2014). |
| Species extinction | The diversity of plant and animal species is essential for a stable environment. Many animal species (insects, birds, mammals, etc.) are important for functioning material cycles, clear water and clean air. Too great a loss of biological diversity would therefore also have serious consequences for humans. Nature conservation organisations say that we are currently experiencing a mass extinction in terms of species diversity. Calculations by the World Wildlife Fund (WWF) show that numerous animal species are already extinct. | Results of a study that examined insect populations in German nature reserves show that a large proportion of all flying insects in Germany have already died in the last 25 years. According to a forecast, **50%** of all currently known animal species worldwide could be extinct by 2100 (Wilson, 2016). Biodiversity is indispensable for maintaining functioning ecosystems worldwide. As part of such systems of nature, the various animals around us provide, among other things, the basis for our food, clean drinking water, healthy soils and the pollination of crops by insects. |
| Deadly heat waves | Like all mammals, humans must constantly regulate their body temperature. Too high a body temperature therefore has dangerous effects on our cardiovascular system and can even be fatal. Heat waves can therefore have serious health consequences. | If human emissions of greenhouse gases continue to rise, the proportion of the world's population that will be exposed to deadly heat waves on at least 20 days a year by 2100 will be **74%** (Eis et al., 2011; Mora et al., 2017). Direct health consequences of such heat waves can be, for example, heat cramps, heat exhaustion or heat stroke. |
| Increase in carbon dioxide and malnutrition | An increase in carbon dioxide in the air causes plants to grow larger. However, plants do not produce more nutrients as a result; on the contrary, they produce more sugar, which takes away space for other important nutrients. The proportion of nutrients such as protein, calcium, iron and vitamin C in some plants decreases sharply as a result. This can lead to iron deficiency in humans, for example. | According to researchers' estimates, the reduction of important ingredients in plants means that by 2050, **18%** of humanity (especially children and women of childbearing age) could be affected by a severely reduced iron intake and possibly other consequences such as anaemia ((Smith et al., 2017). |
| Ocean acidification | Some of the carbon dioxide that drives global warming is absorbed by plants and forests, which use it for photosynthesis. However, another part enters the oceans, where it causes them to acidify. Acidification will continue to increase in the future as carbon dioxide levels in our atmosphere rise. | Even for climate scenarios in which the shift to renewable energies is successful, scientists predict an increase in acidity in the oceans by an average of **39%.** This is particularly harmful for marine life, as they depend on a stable acidity level. Coral reefs, for example, are an important part of the marine ecosystem, affecting weather, climate and oxygen levels in Germany, among other things (IPCC, 2014). |
| Forest and wildfires | Global warming means that drought and heat waves in summer will also increase in Europe. Especially towards the end of the century, we must expect consequences such as forest and wildfires due to rising temperatures. | With a 4°C increase in average temperature, scientists predict a **70%** risk of increased forest and wildfire occurrence in Europe. Such fires would threaten the livelihoods of many Europeans and have far-reaching consequences for the economies of European countries (IPCC, 2014). |
| Floodings | The rise in sea level is steadily increasing the risk of severe flooding, especially in coastal regions and cities. Protective measures are already being taken in many regions of the world to reduce the damage caused by flooding. | With a 4°C increase in the average global temperature, scientists predict an **80%** risk that people in Europe will suffer more damage from river and coastal flooding by the year 2100. Natural disasters such as floods not only often have adverse health consequences through direct physical damage, but also increase the risk of psychological stress such as post-traumatic stress disorder. In addition, river floods are already the natural events that cause the highest economic damage in Germany (Doherty & Clayton, 2011; Hinkel et al., 2014; IPCC, 2014). |
| Droughts in Europe | Droughts are a major challenge for people. They can affect food production and thus people's well-being in the long term. The lowering of groundwater levels, for example, and the drying up of inland waters can affect many areas of our lives. | With a global warming of 3°C and resulting extreme droughts, **67%** of the European population will be affected (Samaniego et al., 2018). Droughts lead to declining labour productivity, rising morbidity rates and deaths due to exposure to heat waves. Farmers, construction workers, children, the homeless and the elderly are particularly at risk (IPCC, 2014). |
| Ozone concentration in the air | Strong solar radiation and high air temperatures in summer lead to increased ozone concentrations in the air. Since ozone is harmful to human health, there are EU guidelines for a concentration value that may not be exceeded on more than 25 days a year. However, global warming is causing a steady increase in the number of days per year in Germany on which this limit value is exceeded. | If temperatures rise by 3°C by 2100, the number of days on which the limit value for the maximum ozone concentration is exceeded could increase by approx. **58%** to 19 days a year. Especially on hot days, the increased ozone concentration can lead to circulatory problems, irritating cough and watery eyes. |
| Losses in fishing | In addition to ocean acidification, heat and overfishing are leading to the mass death of the creatures that live there. By the end of the century, this will greatly reduce the fish catch. | Due to the decline of living creatures in the oceans, the catch potential could collapse by up to **24%** by the end of the century, according to forecasts. The prices of a large number of popular edible fish would rise as a result, and some of these fish species would probably soon no longer exist at all (IPCC, 2014; Müller-Jung, 2019). |
| Melting snow in the Alps | The Alps are the highest high mountain range in Europe. Due to increasing global warming, glaciers and large areas of snow are melting, thus changing the prevailing climate there. This means that an important part of the freshwater reserves of many Europeans is being lost and, in addition, the likelihood of dangerous natural events such as landslides, mudslides and floods is increasing. | With a global warming of 4°C, **70%** of the snow in the Alps will have disappeared by the end of the century. As a result, many Europeans would have to fear for their water supply and could become victims of dangerous natural events. The consequences will also be felt in this country, because Germany depends on the drinking water supply from the mountains (Lynas, 2007; Sauter et al., 2010). |
| Snow days in the Black Forest | The Black Forest in south-western Baden-Württemberg is Germany's highest and largest contiguous low mountain range. Global warming has an influence on the number of snow days there. | The number of snow days in the Black Forest could be reduced by **66%** by the middle of the 21st century. Especially the tourism industry, which has a great economic power in this area, would suffer from the decrease in snow days. Snow is a significant aspect for tourism in the Black Forest. Significantly fewer snow days and corresponding consequences for tourism are also expected for other regions in Germany. (LUBW, 2016; Sauter et al., 2010). |
| Water scarcity | Due to global warming, glaciers in the Himalayas and the Alps are endangered. Freshwater lakes are shrinking or becoming an attractive habitat for bacteria due to rising water temperatures. This leads to an increasing scarcity of clean water, which will increasingly affect people worldwide in the coming decades. | According to projections, **42%** of all people could live in a country by 2030 where there will not be enough water for agriculture, industry and private water supply. Germany would also be directly affected by this, as drinking water is obtained from the Alps, among other places. Water scarcity is also forcing people worldwide to leave their homes and flee as climate refugees to countries like Germany (Doherty & Clayton, 2011). |
| Hurricanes | Environmental researchers predict an increase in extreme weather events and natural disasters as a result of climate change. The number of hurricanes will also continue to rise. | The number of hurricanes in the two highest categories could increase by about **27%** if the Earth warms by 1°C (Holland & Bruyère, 2014). Natural disasters such as hurricanes are associated with considerable health risks, not only as a result of direct physical damage, but also in the form of psychological stress (such as post-traumatic stress disorder). |
| European wetlands | Wetlands are habitats whose plant and animal communities depend on the presence of water (such as ponds, bogs, etc.). They are considered the most productive ecosystems on earth and are able to transform pollutants into essential nutrients. These habitats are highly endangered by climate change-induced dry periods. | If no conservation measures are taken, about **35%** of Europe's wetlands will have disappeared by 2080 (Brown et al., 2011). Nearly half of all animal species live in wetlands or use this space for reproduction. In addition, coastal ecosystems such as wetlands protect coasts from storm damage and flooding. Accordingly, many animal species, as well as humans, are endangered by the disappearance of wetlands. |

**Study 1**

**Measures**

***Full list of the GEB items***

1. For the journey to work or school I use the bike, public transport or walk.
2. I boycott products from companies that are proven to be harmful to the environment.
3. In winter, I turn my heating down if I leave my flat for more than 4 hours.
4. When planning my holiday, I try to avoid travelling by plane.
5. I wash laundry at low temperatures.
6. I make sure to avoid meat products in my diet.
7. When driving, I am committed to keeping fuel consumption as low as possible.

***Defensive Self-Protection***

The Climate Self-Protection Scale (CSPS) was used to assess self-protective emotional and cognitive strategies in relation to climate-related behaviour (Wullenkord & Reese, 2021). The scale consists of a total of 24 items, loading on five factors: (1) rationalisation of one's own involvement, (2) avoidance, (3) denial of the extent of the outcome, (4) denial of the global extent of the outcome, and (5) denial of guilt. The statements were rated on a seven-point Likert scale from *strongly disagree* to *strongly agree*. Cronbach’s alpha of the CSPS was .87 in the present sample.

***Perceived Personal Responsibility***

The degree to which participants felt responsible to mitigate climate change through personal action was assessed with a self-developed new 13-item scale. The items assess the extent to which participants believe they ought to engage in pro-environmental behaviour (e.g., “I am co-responsible to mitigate climate change through personal action”) and the extent to which people couple their own PEB with the condition that others do the same (e.g., “I will only engage in pro-environmental behaviour when a majority of society does so” – reversely scored; for a list of all items, see below). All items were rated on a seven-point Likert scale from *strongly disagree* to *strongly agree*. High values reflect a higher sense of responsibility. Cronbach’s alpha of this scale was .86.

1. I will only change my environmental behaviour when politicians take more committed action against climate change.
2. I will actively protect the climate with my behaviour, even if my environment does not.
3. If other people continue to behave in a climate-damaging way (e.g., by travelling by plane or eating meat), I will do the same.
4. I will only engage in pro-environmental behaviour when a majority of society does so.
5. I will separate my own rubbish even if my neighbours don't.
6. When I see how calmly influential people deal with climate change, I also worry less about the issue.
7. I realise that large parts of the world are affected by climate change.
8. I consider the effects of climate change to be alarming.
9. Urgent action is needed to mitigate the consequences of climate change.
10. The responsibility for effective climate-relevant change lies solely in the hands of politicians.
11. The responsibility for effective climate-relevant change lies in my hands, among others.
12. I know what I can do to help limit climate change.
13. I decide to make a contribution to the to limit climate change.

***Self-Efficacy***

Climate change-related self-efficacy – that is, the belief that one can contribute to climate change mitigation through personal action – was assessed by eight items derived from the recent Triple-A framework (Hamann et al., 2024). The items (e.g., “If I want to, I can organise my future travels without flights”) were rated on a seven-point Likert scale from *strongly disagree* to *strongly agree*. High values reflect higher self-efficacy beliefs. Cronbach’s alpha of this scale was .69 in the current sample.

***Perceived Threat***

Perceived threat of climate change was assessed using a single item rated on a sliding scale from 0% = *not at all threatening* to 100% = *extremely threatening*).

**Additional Results**

**Intercorrelations**

We first explored the correlations among belief updating, PEB, and other psychological factors (see supplementary Table S2).

Table S2

*Correlational analyses*

|  | Defensive self-protection | Personal responsibility | Self-efficacy | Perceived threat | Pro-environmental behaviour |
| --- | --- | --- | --- | --- | --- |
| Asymmetry in belief updating | -.211* | .047 | -.116 | -.224* | .041 |
| Defensive self-protection | - | -.650*** | -.270** | -.194* | -.327*** |
| Personal responsibility | - | - | .406*** | .446*** | .481*** |
| Self-efficacy | - | - | - | .347*** | .360*** |

*Note: *p* < .05, ** *p* < .01, *** *p* <.001. An asymmetry in belief updating score > 0 reflects a greater integration of good news, whereas a score < 0 reflects a greater integration of bad news.

**Preregistered analysis to predict PEB**

A hierarchical linear regression indicated that self-protection, personal responsibility, and self-efficacy explained 26.4% of the variance in PEB (see Table S3). Adding belief updating as a predictor did not significantly add explained variance.

Table S3

*Hierarchical regression analysis predicting pro-environmental behaviour*

|  |  | **Criterion: pro-environmental behaviour** | | | |
| --- | --- | --- | --- | --- | --- |
| **Model** | **Predictors** | **β** | ***R*^2^** | ***∆R²*** | ***∆F*** |
| Step 1 |  |  | .264 | .264*** | 12.572*** |
|  | Self-protection | -.022 |  |  |  |
|  | Personal responsibility | .387** |  |  |  |
|  | Self-efficacy | .197* |  |  |  |
| Step 2 |  |  | .266 | .002 | 0.257 |
|  | Self-protection | -.008 |  |  |  |
|  | Personal responsibility | .391** |  |  |  |
|  | Self-efficacy | .205* |  |  |  |
|  | Asymmetry in belief updating | -.044 |  |  |  |

*Note*: * *p* < .05, ** *p* < .01, *** *p* < .001. An asymmetry in belief updating score > 0 reflects a greater integration of good news, whereas a score < 0 reflects a greater integration of bad news.

**Study 2**

**Additional measures**

**Intentions for Pro-Environmental Behaviour**

Like previous research (Kube et al., 2024), we assessed people’s intentions to engage in more pro-environmental actions using an 8-item questionnaire adapted by Broomell et al., (2015). Four items assess the general intention to do more to mitigate climate change (e.g., “I will strive to do something about the negative consequences of climate change”), whereas another four items assess the intention for behaviours (e.g., “I plan to fly less to protect the climate”). Participants completed this scale at the beginning, after the video-based interventions at the end of the entire baseline assessment (i.e., post-intervention), and four weeks later (i.e., 4-week follow-up). Cronbach’s alpha of the scale was .87 at baseline, .86 after the end of the first assessment, and .86 four weeks later.

**Perception of Threat and Anxiety in Relation to Climate Change Events**

Before beginning with the belief update task, participants indicated the degree to which they perceived climate change as threatening and anxiety-provoking. When doing so, we differentiated between asking participants whether they generally and personally perceived climate change to be threatening/anxiety-provoking. This distinction drew from previous research showing that people’s personal risk estimates strikingly differ from how likely they think others will be affected by the respective risk (Globig et al., 2022; Kuper-Smith et al., 2021). When assessing personal perception of threat and anxiety, we asked participants to think about their own future and about people close to them. Each of the four items (threatening – general and personal; anxiety-provoking – general and personal) were rated on a scale from 0% (*not at all threatening/anxiety provoking*) to 100% (*absolutely threatening/anxiety-provoking*). Subsequently, participants indicated the degree to which they perceived each of the 20 climate change scenarios from the belief update task as threatening/anxiety-provoking, using the four items (e.g., "To what extent do you personally feel threatened by this event? Please refer to the possible threat you feel in relation to your own future or that of people close to you.").

**Depressive Symptoms**

Depressive symptoms were assessed using the well-established Patient Health Questionnaire (PHQ-9), which is also suitable for the general population (Kroenke et al., 2001). We assessed depressive symptoms because previous research has shown that the optimistic update bias, which is typically found in healthy people, is absent in people with depression. Accordingly, we wanted to explore whether depressive symptoms relate to the processing of good news vs. bad news in relation to climate change, which is reported in the supplement. The PHQ-9 has 9 items which are rated on a 4-point scale (0 to 3), such that the sum score can range from 0 to 27. A PHQ-9 sum score of *M* = 7.32 (*SD* = 5.22) indicated that participants from the current sample had on average mild depressive symptoms according to the classification by Kroenke et al. (2001).

**Additional results**

**Association of Belief Updating with Perceived Threat and Anxiety**

In trials 1-10, the asymmetry in belief updating was significantly associated with the perception of the climate change events as generally threatening (*r* = -.128, *p* = .002) and personally threatening (*r* = -.144, *p* < .001), with greater threat being associated with a greater integration of bad news over good news. Similarly, the perception of the climate change events as generally (*r* = -.130, *p* = .001) and personally anxiety-provoking (*r* = -.149, *p* < .001) was significantly associated with a greater integration of bad news over good news. When considering all 20 trials, all correlations remained significant and were even slightly higher in size (-.145 ≥ *r* ≤ -.160).

**Additional Effects of the Video-Based Interventions**

In some additional non-preregistered analyses, we explored whether the video-based interventions had any effects on valence-dependent belief updating and the accuracy of risk perceptions.

***Change in Valence-Dependent Belief Updating***

A repeated-measures ANOVA indicated a significant main effect of time, *F*(1, 592) = 11.799, *p* < .001, η_p_^2^ = .020, 95% CI [.004, .047], showing that across conditions, participants’ tendency to integrate good news about climate change more than bad news from the first 10 trials, significantly declined in the subsequent trials 11-20, resulting in a more balanced integration of good news and bad news. The time by condition interaction was not significant, *F*(3, 592) = 1.857, *p* = .136, η_p_^2^ = .009, 95% CI [0, .026], although descriptively the decline was greatest in the *Threat* and *Threat + Options for Action* condition (see supplementary Figure S2).

Asymmetry in belief updating

**Figure S1.** Results for change in group differences in valence-dependent belief updating. Positive values reflect an integration of good news over bad news and negative values a greater integration of bad news, respectively. Error bars reflect the standard error of the mean.

***Change in Pro-Environmental Intentions***

The repeated-measures ANOVA indicated a significant main effect of time, *F*(1.936, 780.237) = 93.635, *p* < .001, η_p_^2^ = .189, 95% CI [.141, .235]. As can be seen in Figure S2, participants’ pro-environmental intentions increased from baseline (*M* = 5.59, *SD* = 0.97) to after watching the videos (*M* = 5.86, *SD* = 0.94), before they re-decreased at follow-up four weeks later (*M* = 5.55, *SD* = 0.96). The time by condition interaction was not significant, *F*(5.808, 780.237) = 0.822, *p* = .550, η_p_^2^ = .006, 95% CI [0, .013], nor was the main effect of condition, *F*(3, 403) = 0.668, *p* = .572, η_p_^2^ = .005, 95% CI [0, .020].


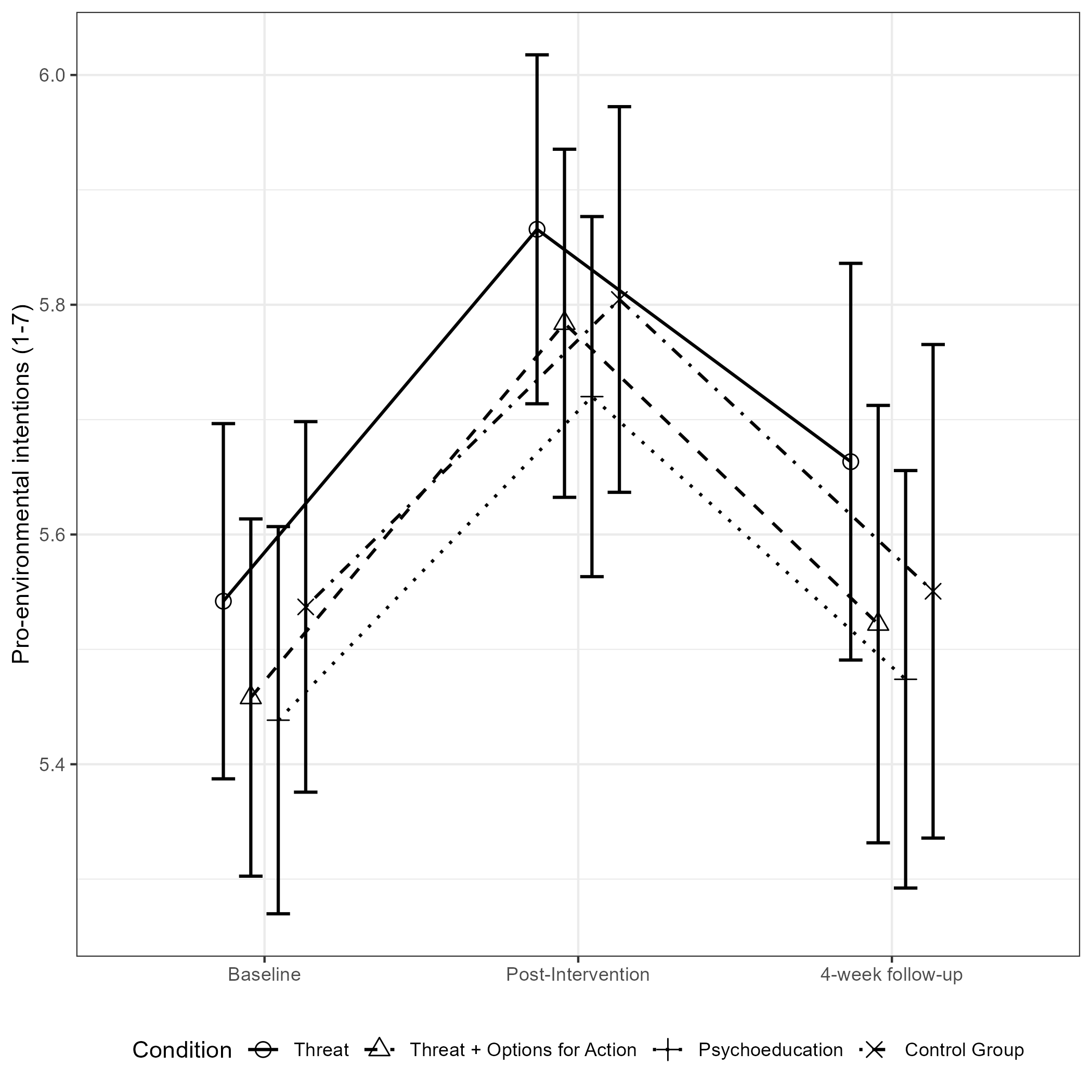


**Figure S2.** Illustration of the results for changes in intentions for pro-environmental behaviour. Error bars reflect the 95% confidence interval.

The pre-registered inclusion of gender led to a significant time by gender interaction, *F*(1.935, 772.088) = 3.104, *p* = .047, η_p_^2^ = .008, 95% CI [.001, .023], indicating that the temporary increase in intentions from baseline to post-intervention was greater in women than in men, *t*(602) = -2.209, *p* = .028, *d* = -0.207, 95% CI [-.391, -.023]. From post-intervention to the 4-week follow-up, the decline in pro-environmental intentions was not different between women and men, *t*(602) = -1.186, *p* = .236, *d* = -0.141, 95% CI [-.373, -.092]. A significant main effect of gender indicated that women had overall more pro-environmental intentions than men, *F*(1, 399) = 5.917, *p* = .015, η_p_^2^ = .015, 95% CI [.001, .046].

***Change in the Accuracy of Risk Estimation***

A repeated-measures ANOVA indicated a significant main effect of time, *F*(1, 600) = 79.594, *p* < .001, η_p_^2^ = .117, 95% CI [.073, .166], showing that across conditions, the difference between participants’ first risk estimations for the climate change events and the actual prognosis (i.e., the estimation error) decreased from trials 1-10 to trials 11-20. In other words, people became more accurate in predicting the likelihood of the climate change events. The time by condition interaction was not significant, *F*(3, 600) = 0.787, *p* = .502, η_p_^2^ = .004, 95% CI [0, .015], nor was the main effect of condition, *F*(3, 600) = 0.806, *p* = .491, η_p_^2^ = .004, 95% CI [0, .015].

For changes in the accuracy of participants’ risk estimation, we examined gender differences in an exploratory manner. A significant main effect of gender indicated that overall women were better than men at accurately predicting the likelihood of climate change events, *F*(1, 596) = 5.749, *p* = .017, η_p_^2^ = .010, 95% CI [.001, .031]. This effect was driven by a higher accuracy of women in the first 10 trials, *t*(602) = 2.430, *p* = .015, *d* = 0.228, 95% CI [0.044, 0.412], whereas men and women did not differ in their accuracy in trials 11-20, *t*(602) = 1.526, *p* = .127, *d* = 0.143, 95% CI [-.041, .327].

A significant time by condition by gender interaction, *F*(3, 596) = 2.956, *p* = .032, η_p_^2^ = .015, 95% CI [.001, .035], indicated that in the *Threat* condition, men’s accuracy, unlike women’s accuracy, did not improve, whereas in in all other conditions, women improved their risk estimation less than men (see Figure S3).


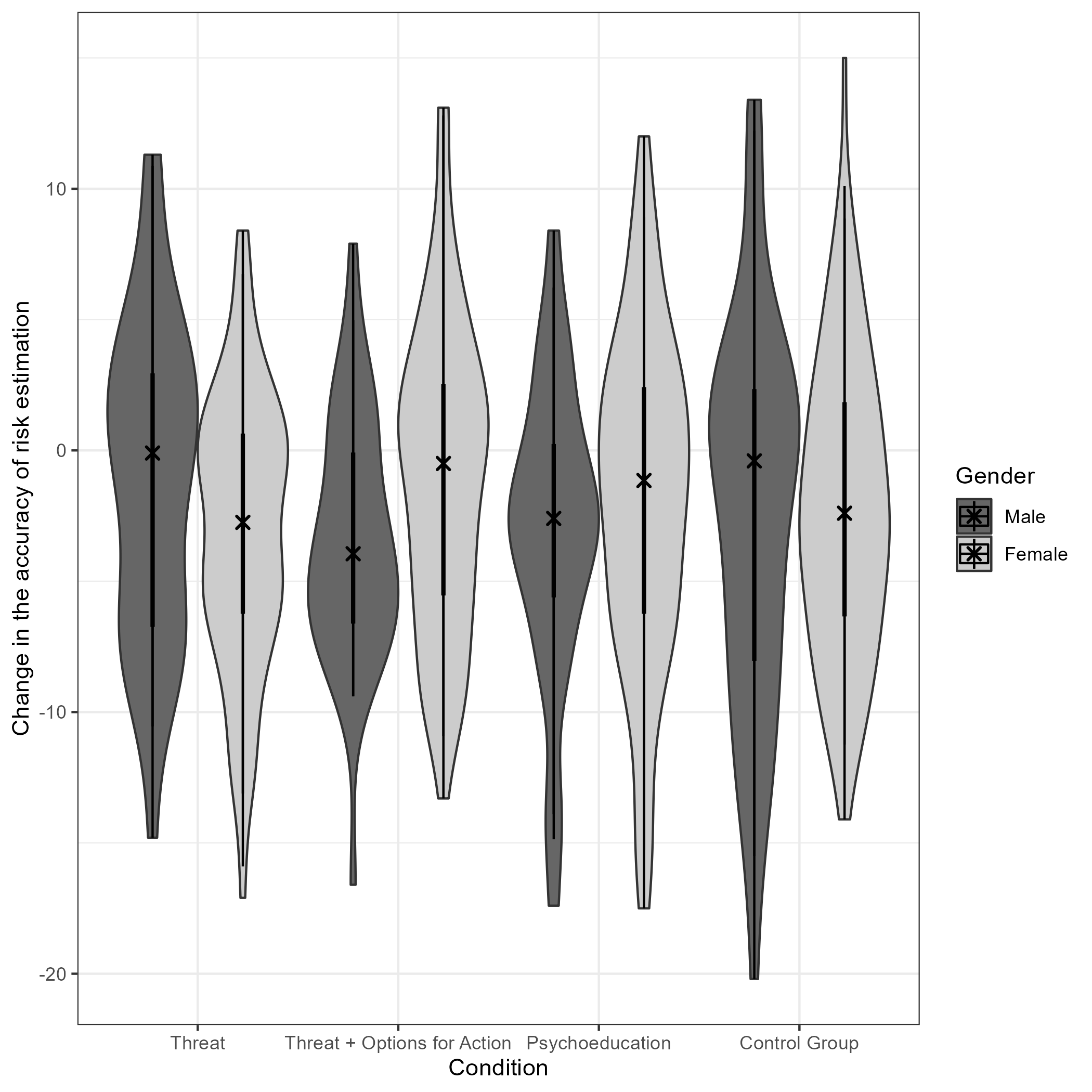


***Figure S3.*** *Violin plot for gender differences in the improvement in risk perception from trials 1-10 to trials 11-20. The more negative the values depicted, the greater the improvement. The x reflects the median. Results show that in the condition, in which only a video about the threats of climate change was shown, men did not improve in their accuracy of their risk perception. In the other experimental conditions, they improved more than women.*

**Association of depressive symptoms with belief updating**

According to a correlation analysis, depressive symptoms as assessed with the PHQ-9 were unrelated to the integration of good news relative to bad news, *r* = -.068, *p* = .096. Depressive symptoms did correlate though with a greater perception of the climate change events as generally threatening (*r* = .123, *p* = .002), personally threatening (*r* = .161, *p* < .001), generally anxiety-provoking (*r* = .121, *p* = .003), and personally anxiety-provoking (*r* = .182, *p* < .001).

# References

Broomell, S. B., Budescu, D. V., & Por, H.-H. (2015). Personal experience with climate change predicts intentions to act. *Global Environmental Change*, *32*, 67–73.

Faul, F., Erdfelder, E., Lang, A.-G., & Buchner, A. (2007). G*Power 3: A flexible statistical power analysis program for the social, behavioral, and biomedical sciences. *Behavior Research Methods*, *39*, 175–191.

Globig, L. K., Blain, B., & Sharot, T. (2022). Perceptions of personal and public risk: Dissociable effects on behavior and well-being. *Journal of Risk and Uncertainty*, *64*(2), 213–234.

Hamann, K. R. S., Wullenkord, M. C., Reese, G., & van Zomeren, M. (2024). Believing that we can change our world for the better: A Triple-A (agent-action-aim) framework of self-efficacy beliefs in the context of collective social and ecological aims. *Personality and Social Psychology Review*, *28*(1), 11–53. https://doi.org/10.1177/10888683231178056

Hoffmann, T., Ye, M., Zino, L., Cao, M., Rauws, W., & Bolderdijk, J. W. (2024). Overcoming inaction: An agent-based modelling study of social interventions that promote systematic pro-environmental change. *Journal of Environmental Psychology*, *94*, 102221. https://doi.org/10.1016/j.jenvp.2023.102221

Kaiser, F. G., Byrka, K., & Hartig, T. (2010). Reviving Campbell’s paradigm for attitude research. *Personality and Social Psychology Review*, *14*(4), 351–367.

Kaiser, F. G., Hartig, T., Brügger, A., & Duvier, C. (2013). Environmental protection and nature as distinct attitudinal objects: An application of the Campbell paradigm. *Environment and Behavior*, *45*(3), 369–398.

Kaiser, F. G., & Wilson, M. (2004). Goal-directed conservation behavior: The specific composition of a general performance. *Personality and Individual Differences*, *36*(7), 1531–1544.

Kroenke, K., Spitzer, R. L., & Williams, J. B. (2001). The PHQ‐9: Validity of a brief depression severity measure. *Journal of General Internal Medicine*, *16*(9), 606–613.

Kube, T., Wullenkord, M., Rozenkrantz, L., Kramer, P., Lieb, S., & Menzel, C. (2024). How people update their beliefs about climate change: An experimental investigation of the optimistic update bias and how to reduce it. *Political Psychology*, *45*(1), 175–192. https://doi.org/10.1111/pops.12920

Kuper-Smith, B. J., Doppelhofer, L. M., Oganian, Y., Rosenblau, G., & Korn, C. W. (2021). Risk perception and optimism during the early stages of the COVID-19 pandemic. *Royal Society Open Science*, *8*(11), 210904. https://doi.org/10.1098/rsos.210904

Umweltbundesamt. (2021). *Treibhausgasemissionen sinken 2020 um 8,7 Prozent*. https://www.umweltbundesamt.de/presse/pressemitteilungen/treibha usgasemissionen-sinken-2020-um-87-prozent

Wullenkord, M. C., & Reese, G. (2021). Avoidance, rationalization, and denial: Defensive self-protection in the face of climate change negatively predicts pro-environmental behavior. *Journal of Environmental Psychology*, *77*, 101683.
